# Biodegradable Nanofibrous Membranes for Medical and Personal Protection Applications: Manufacturing, Anti-COVID-19 and Anti-Multidrug Resistant Bacteria Evaluation

**DOI:** 10.3390/ma14143862

**Published:** 2021-07-10

**Authors:** Latifah Abdullah Alshabanah, Mohamed Hagar, Laila A. Al-Mutabagani, Ghada M. Abozaid, Salwa M. Abdallah, Hoda Ahmed, Ahmed H. Hassanin, Nader Shehata

**Affiliations:** 1 Chemistry Department, College of Science, Princess Nourah Bint Abdulrahman University, Riyadh 11671, Saudi Arabia; laalsabanah@pnu.edu.sa (L.A.A.); laalmutbagani@pnu.edu.sa (L.A.A.-M.); 2 Chemistry Department, College of Sciences, Taibah University, Yanbu 30799, Saudi Arabia; ahoda@sci.cu.edu.eg; 3 Chemistry Department, Faculty of Science, Alexandria University, Alexandria 21321, Egypt; 4 Pharmaceutical Practice Department, College of Pharmacy, Princess Nourah Bint Abdulrahman University, Riyadh 11671, Saudi Arabia; Gaabozeed@pnu.edu.sa; 5 Mammalian and Aquatic Toxicology Department, Central Agricultural Pesticides Lab (CAPL), Agricultural Research Center (ARC), Giza 12611, Egypt; salwaabdallah17@gmail.com; 6 Department of Chemistry, Faculty of Science, Cairo University, Cairo 12613, Egypt; 7 Center of Smart Materials Nanotechnology and Photonics (CSMNP), Smart CI Research Centre, Alexandria University, Alexandria 21544, Egypt; ahassanin2003@yahoo.com (A.H.H.); nader83@vt.edu (N.S.); 8 Materials Science & Engineering Department, School of Innovative Design Engineering, Egypt-Japan University of Science and Technology (E-JUST), New Borg El-Arab City, Alexandria 21934, Egypt; 9 Department of Textile Engineering, Faculty of Engineering, Alexandria University, Alexandria 21544, Egypt; 10 Department of Engineering Mathematics and Physics, Faculty of Engineering, Alexandria University, Alexandria 21544, Egypt; 11 USTAR Bio Innovations Centre, Faculty of Science, Utah State University, Logan, UT 84341, USA; 12 Department of Physics, School of Engineering, Kuwait College of Science and Technology (KCST), Doha Superior Rd., Jahraa 13133, Kuwait

**Keywords:** biodegradable nanofibrous membrane, PVA-ZnO or -CuO nanohybrids, anti-COVID-19 nanofibers, anti-multi-drug resistant bacterial nanofibers

## Abstract

Biodegradable nanofibrous hybrid membranes of polyvinyl alcohol (PVA) with ZnO and CuO nanoparticles were manufactured and characterized, and their anti-COVID-19 and anti-multidrug resistant bacteria activities were also evaluated. The morphological structures of the prepared PVA composites nanofibers were observed by scanning electron microscope (SEM), which revealed a homogenous pattern of the developed nanofibers, with an average fibrous diameter of 200–250 nm. Moreover, the results of the SEM showed that the fiber size changed with the type and the concentration of the metal oxide. Moreover, the antiviral and antibacterial potential capabilities of the developed nanofibrous membranes were tested in blocking the viral fusion of SARS-COV-2, as a representative activity for COVID-19 deactivation, as well as for their activity against a variety of bacterial strains, including multi-drug resistant bacteria (MDR). The results revealed that ZnO loaded nanofibers were more potent antiviral agents than their CuO analogues. This antiviral action was attributed to the fact that inorganic metallic compounds have the ability to extract hydrogen bonds with viral proteins, causing viral rupture or morphological changes. On the other hand, the anti-multi-drug resistant activity of the prepared nanofibers was also evaluated using two techniques; the standard test method for determining the antimicrobial activity of immobilized antimicrobial agents under dynamic contact conditions and the standard test method for determining the activity of incorporated antimicrobial agents in polymeric or hydrophobic materials. Both techniques proved the superiority of the ZnO loaded nanofibers over the CuO loaded fibers. The results of the antiviral and antibacterial tests showed the effectiveness of such nanofibrous formulas, not only for medical applications, but also for the production of personal protection equipment, such as gowns and textiles.

## 1. Introduction

The COVID-19 pandemic has forced the global population to organize new ways of living, including the wearing of better protective equipment as a new norm. The design of new protective personal equipment (PPEs) based low-cost materials offers better protection for users against airborne pollutants and pathogens [1]. Recent investigations have shown new advances in the development of materials with improved filtering capacity and antimicrobial activity [2,3]. Moreover, the wearing of PPE is a key strategy for airborne disease prevention that cannot be easily substituted [4].

Today, the development of safe and low-cost natural biodegradable composite nanofibers as antimicrobials and antivirals, including COVID-19 resistance, for environmental remediation could be of great interest because of their non-toxic nature [5,6]. In addition, biodegradable properties in the choice of polymers could add better characteristics to protective personal equipment (PPE), due to their natural aspects [7]. Designing such approaches begins to address the PPE challenges of the healthcare workplace. Moreover, most reports have focused on how to design unique healthcare personnel PPE for communicating with patients [8,9]. Antibacterial products will be essential in the future because of their impact against coronavirus, including the use of safe and hygienic products with antimicrobial characteristics. Today, nano-materials are found in a broad range of existing products, such as in electronics, health, paints, food, and clothing [10,11,12]. Moreover, medicine is among the areas with a growing interest in the use of nanotechnology. Investigations into the application of nanotechnology for preparing appropriate antibacterial derivatives based on many metallic and oxide nano-materials have recently been reported [13]. Researchers have tried to produce a multitude of materials [14] for use in all domains needing efficient antibacterial products. It was reported that antibacterial nanoparticles should be added to the commonly used pigments for coating or in some clay minerals, such as vermiculite and montmorillonite, as well as in composite products to be added at the stage of surface sizing [13].

On the other hand, nano-materials can interact with the viral particle or the surface proteins, leading to the inactivation of viruses. The properties of antiviral nano-materials, their potential mechanisms of action, ongoing preclinical/clinical investigations, and currently approved products for consumer use are discussed below [15,16].

Antiviral textiles are textiles that can reduce the number of infective virus particles that come into contact with the textile’s surface. This standard establishes a quantitative method for evaluating the antiviral effectiveness of such items. As health knowledge has grown, people have begun to pay attention to the removal or minimization of harmful pathogenic organisms from the atmosphere or surfaces; as a result, antimicrobial materials of various types are being developed. Many scientists have recently looked into antibacterial, antifungal, and anti-yeast fabrics, including textiles. A number of studies have been performed so far to establish antimicrobial and antiviral textile drugs, but little progress has been achieved [17].

One of the most important aspects to take into consideration is the safety of the nanomaterial. Based on the current weight of evidence from the available data, the risk for humans from the use of the nano-structural ZnO or CuO currently used in preparations with direct skin contact is considered negligible [17,18]. The dermal route of exposure for ZnO in typical sunscreen formulas is considered safe, since there is neither proof of penetration into the viable epidermis nor toxicity issues [19]. However, there are still uncertainties regarding the possibility of nanomaterials penetrating through the stratum corneum into viable layers, where toxicological concerns may arise [20].

Concerning CuO NPs, very few studies have investigated the toxic action of CuO NPs towards humans, with a special focus on the skin penetration route of entry. Using a Fanz static diffusion cell model, experimental data showed that the CuO NP absorption through intact skin was negligible. However, with damaged skin, the increasing permeation of copper would justify the capacity of CuO NPs to pass the first skin layers and release Cu^2^^+^ ions into the stratum corneum, reaching the receptor fluid in the in vitro diffusion cell system. 

The objectives of this research work were to develop an antibacterial and antiviral membrane by electrospinning a biodegradable PVA polymer and two metal oxides (ZnO and CuO). Moreover, the effectiveness of such nanofibrous formulas, not only in medical applications, but also in the production of everyday textiles and technical textiles was also investigated by evaluation of their biological activities for blocking viral fusion of SARS-COV-2, as a representative activity of COVID-19, as well as their action on multi-drug resistant bacterial strains.

## 2. Material and Methods

### 2.1. Materials

Polyvinyl alcohol (PVA) pellets (Mw = 205,000 g/mol, Sigma Aldrich, St. Louis, MO, USA) were acquired to prepare 10 wt% of PVA solution. ZnO nanoparticles (<100 nm particle size) were supplied by Sigma Aldrich, St. Louis, MO, USA, along with copper sulfate (Sigma Aldrich) NaOH (Sigma Aldrich) and zinc nitrate hexa-hydrate (Sigma Aldrich).

### 2.2. Synthesis of CuO Nanoparticles 

A 0.4 M aqueous solution of copper sulfate (1.00 g copper sulfate in 10 mL deionized water) (CuSO_4_·5H_2_O) and 2.0 M aqueous solutions of sodium hydroxide (NaOH) was prepared in distilled water (1.25 g NaOH in 10 mL distilled water). Then the solution of copper sulfate was heated to 85 °C and kept under constant stirring using a magnetic stirrer till complete dissolving of the copper sulfate. After 15 min, sodium hydroxide solution (2.0 M) was added as a reducing agent, the black precipitate formed was Cu(OH)_2_. This precipitate was filtered out and washed with distilled water and ethanol several times alternatively to remove the impurities. After washing, the precipitate was kept in an oven at 200 °C for 3 h. During this process, all copper hydroxide was converted to copper oxide [21].

### 2.3. Manufacturing of Nanofibrous Membranes

Polyvinyl alcohol (PVA) pellets were used to prepare 10 wt% of PVA solution. The PVA pellets were added to distilled water at 70 °C for 1 h and left on the stirrer overnight at room temperature. ZnO nanoparticles (<100 nm particle size) were added with different concentrations (5, 7, and 9 wt%) to the PVA solution and stirred overnight. Similar concentrations of CuO nanoparticles (5, 7 and 9%) were added to the PVA solution and prepared for electrospinning through continuous stirring and sonication; the particle size was 6–13 nm from the spectral data of CuO nanoparticles, however, ZnO particle size was 10 nm (see supplementary data, Figures S1–S5). 

The prepared solutions were electrospun by adding 5 mL of each into a plastic syringe with stainless-steel needle of gauge 18. A high-voltage power supply CZE1000R (Spellman, Hauppauge, NY, USA) was connected and provided positive voltage of 25 kV to the needle. A feed rate of 1 mL/h was set using a syringe pump NE1000 (New Era Pump Systems, Suffolk County, NY, USA). The distance between the needle tip and the grounded rotating collector was adjusted to 10 cm.

### 2.4. Fiber Morphological and Physical Characterizations

The morphological structures of the PVA composite nanofibrous membranes were observed by scanning electron microscope (JEOL, JSM-6010LV-SEM, Tokyo, Japan). One sample for each concentration was cut and stacked onto a carbon tab before sputtering with platinum. The average fiber diameter and fiber diameter distribution were obtained by using Image-J software (Madison, WI, USA) at three different image scales (1 µm, 5 µm, and 10 µm).

A Fourier transform infrared spectrometer (FT-IR) (Vertex 70 FT-IR, Bruker, Billerica, MA, USA) was operated in ATR mode. Samples were scanned 120 times at a resolution of 5 cm^−1^ over a range of 4000–400 cm^−1^.

### 2.5. Antibacterial Activity

#### 2.5.1. Microorganisms

*Staphylococcus aureus ATCC 6538* and *Klebsiella pneumoniae ATCC 4352* were kindly provided by Naval Medical Research Unit (NAMRU) No. 3 in Cairo, Egypt. While Multidrug resistant (MDR) strains, namely Methicillin resistant *S. aureus* (MRSA), Methicillin resistant *S. epidermidis* (MRSE), *P. aeruginosa,* and *K. pneumoniae,* were kindly provided by the Surveillance Microbiology Department’s strain bank from Alexandria University main hospital, Alexandria.

#### 2.5.2. Multi-Drug Resistant Antibacterial Activity of the Prepared Nanofibers

***Qualitative method,*** Muller Hinton agar plates were inoculated with the tested microbes, then each of the prepared nanofibrous membranes (PVA-ZnO 5%, PVA-ZnO 7%, PVA-ZnO 9%, PVA-CuO 5%, PVA-CuO 7%, and PVA-CuO 9%) were cut to approximately 1 cm^2^ and individually placed on the surface of the inoculated agar plates. PVA nanofibers were used as a control [22]. The antibacterial activity was expressed as inhibition zone halos around the tested nanofibers ASTM E2180-18 (standard test method for determining the activity of incorporated antimicrobial agent(s) in polymeric or hydrophobic materials) [23].

***Quantitative method,*** a test was used to evaluate the antibacterial activity of both ZnO and CuO nanofibers according to ASTM E 2149-01 (standard test method for determining the antibacterial activity of immobilized antimicrobial agents under dynamic contact conditions) [24]. The antibacterial functions of the electrospun nanofibrous membranes were expressed as the percentage reduction of test organisms after a specified contact time with the nanofibers and according to the following formula:

(1) $$R\;\left(\%\right)=\frac{B-A}{B}\times 100$$

*R* is the reduction rate of the number of colonies, *A* is the number of bacterial colonies in the flask containing nanofibrous membranes after a specified contact time, and *B* is the number of bacterial colonies in the flask before the addition of nanofibrous membranes. [25].

Results were expressed as mean of three trials ± standard deviation. The means of the treatments were considered significant when 0.05 > *p* > 0.01.

### 2.6. Antiviral Activity of the Prepared Nanofibers

The antiviral activity of the prepared nanofibrous membranes was evaluated according to ISO 18184 with modifications. A SARS-COV-2 inhibitor screening kit (COVID-19 Coronavirus Assay Kit, Biosource, Muskego, WI, USA) was used to evaluate the anti-COVID-19 activity of the developed nanofibers. The proposed inhibitor screening assay was based on a colorimetric ELISA kit, which measures the binding of the RBD of the Spike S protein from SARS-CoV-2 to its human receptor ACE2. Thus, this assay allows identifying and characterizing the effect of different inhibitory compounds on the inhibition of the binding of SARS-CoV-2 virus to human ACE2. According to the sample manual, different concentration of the nanofibers were prepared and incubated with Spike S for one h at 37 °C, then the OD was measured at 450 nm using an ELISA reader. As a result, the damaged virus loses its fit with the receptor of the host cell and this reduces the virus activity [4]. 

## 3. Results and Discussion

### 3.1. Morphological Characterization of Nanofibers

**Figure 1:**
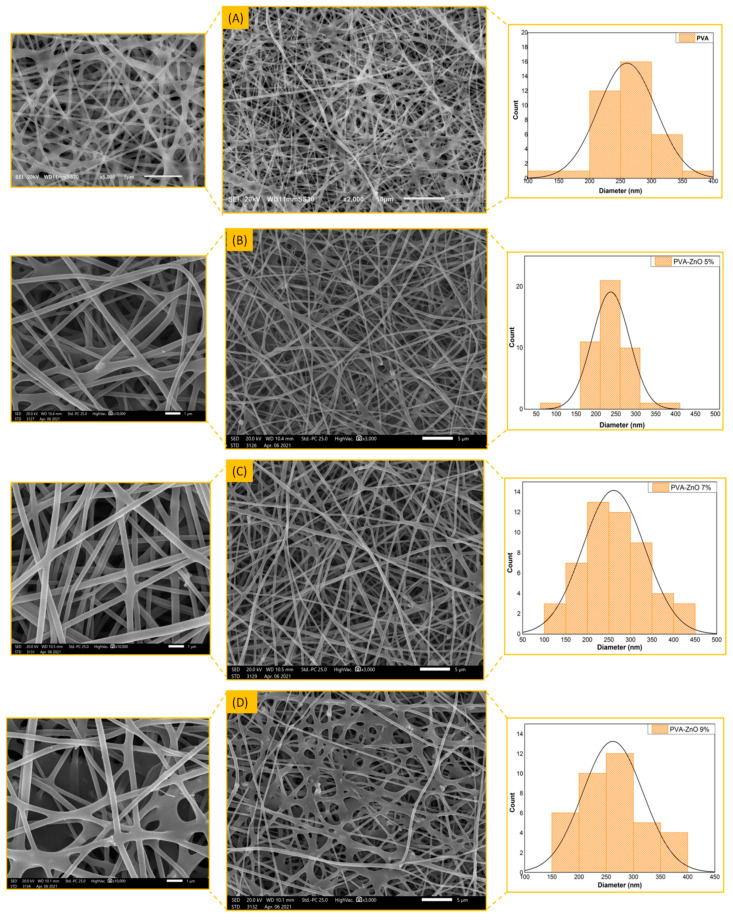

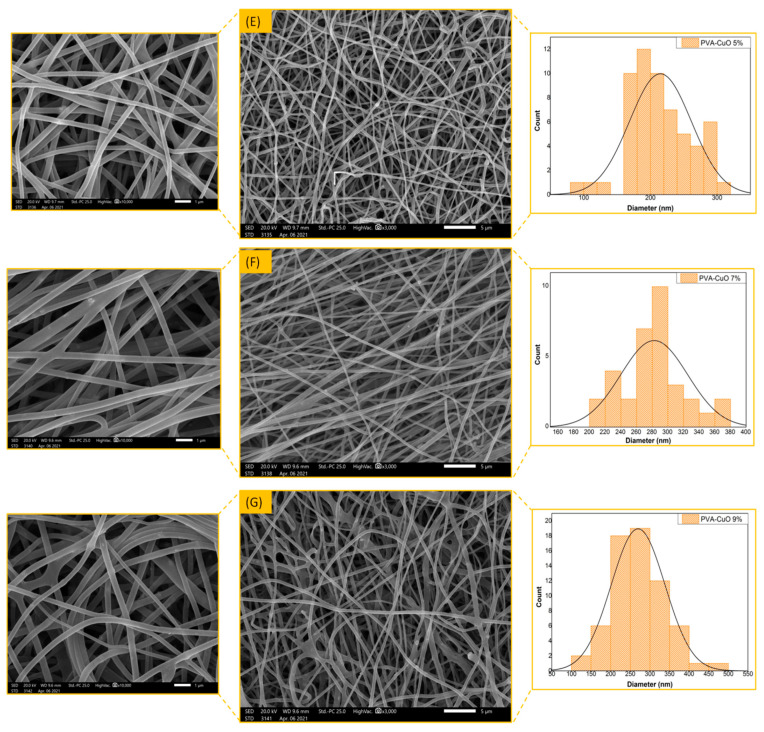
SEM images of PVA (**A**), PVA-ZnO 5% (**B**), PVA-ZnO 7% (**C**), PVA-ZnO 9% (**D**), PVA-CuO 5% (**E**), PVA-CuO 7% (**F**), and PVA-CuO 9% nanofibers (**G**).

The PVA composite nanofibrous membrane morphology was examined by field emission scanning electron microscope (FESEM). The average fiber diameter of the nanofibers was obtained using Image-J software. The SEM images revealed that the nanofibrous membrane had a homogenous structure, with a fine diameter of about 200–250 nm. The addition of nanoparticles did not affect the fiber diameter as a result of the good dispersal, small particle size, and optimized spinning conditions, Figure 1, Table 1.

### 3.2. Physicochemical Characterization of Nanofibers

**Figure 2:**
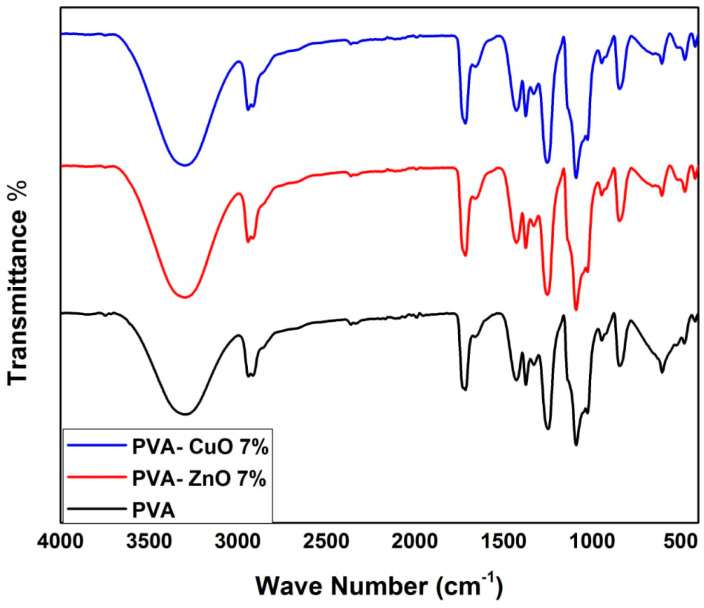
FT-IR of PVA, PVA-ZnO, and PVA-CuO nanofibers.

Figure 2 shows the infrared spectra of the synthesized PVA nanofibers, with and without ZnO and CuO, in the 700–4000 cm^−1^ range. PVA had distinct peaks at 715, 871, 1091, 1431, 1670, 2923, and 3655 cm^−1^, as illustrated in Figure 2. The IR peaks at 715 and 871 cm^−1^ are due to the out of plane vibrations of the O–H and C–H bonds, respectively. The IR peaks at 1091 and 1431 cm^−1^ are caused by C–O stretching and bending vibrations of the CH_2_ group, respectively. An IR peak at 1737 cm^−1^ is caused by the C=O stretching vibrations of the residual non-hydrolyzed vinyl acetate group of the PVA. An IR peak at 2923 cm^−1^ is caused by the stretching vibration of the CH_2_ group. The strong broad peak at 3655 cm^−1^ is assigned to the OH stretching vibrations of strong hydrogen bonds that form intra- and inter-molecule. The increased transmittance of ZnO- and CuO-embedded PVA fibers was accompanied by a considerable effect on the vibrational frequency allocated to in-plane O–H vibrations, being 3447 and 3409 cm^−1^ for ZnO and CuO, respectively. However, the C–H vibrations at 3143 cm^−1^ for the PVA fiber were shifted to 3111 and 3218 cm^−1^ for ZnO and CuO, respectively. Moreover, the C=O stretching vibrations of the residual non-hydrolyzed vinyl acetate group at 1737 cm^−1^ of the pure PVA was also affected by ZnO and CuO, being 1759 and 1743 cm^−1^, respectively. The large peak at a lower wavenumber at 852 cm^−1^ represents out-of-plane O–H vibration and is also another confirmation of the composite formation assigned at 807 and 809 cm^−1^ for ZnO and CuO, respectively [26]. The diagram depicts how the implanted metal oxides interact with the PVA fiber matrix O–H group. Moreover, the increment of the metal oxide amount highly affected the wavenumber of the characteristic peaks. 

### 3.3. Antiviral Activity

**Figure 3:**
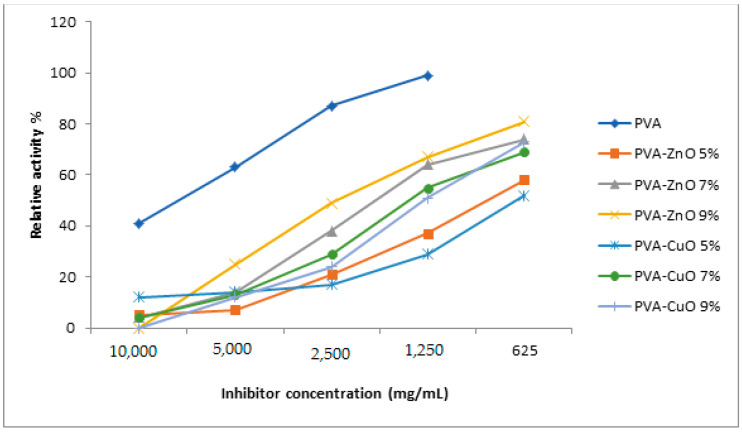
Remaining activity percentage of blocking viral fusion of SARS-COV-2 of the prepared nanofibers.

A few researchers have started to prevent viral infectivity using textile materials, which are usually fibrous structures. Hence, in the present manuscript we aimed to start the journey by taking one step further, using in vitro evaluation against the Spike S-protein from SARS-CoV-2. It was found that by increasing the metal oxide concentration the inhibitory effect was increased. However, ZnO loaded nanofibers were the most potent antiviral agent (Figure 3). The aforementioned activity could be explained by the inorganic metallic substances being able to extract the hydrogen atom in the viral proteins, which leads to viral rupture or morphology changes [27]. 

Imai et al. studied the impact of copper ion-exchanged zeolites on the inactivation of avian influenza viruses (AIVs) on cotton textiles and found that both highly pathogenic H5N1 and low pathogenic H5N3 viruses were inactivated on the copper ion-exchanged zeolite textile materials, even after short incubations [28].

### 3.4. Multi-Drug Resistant Antbacterial Activity

**Figure 4:**
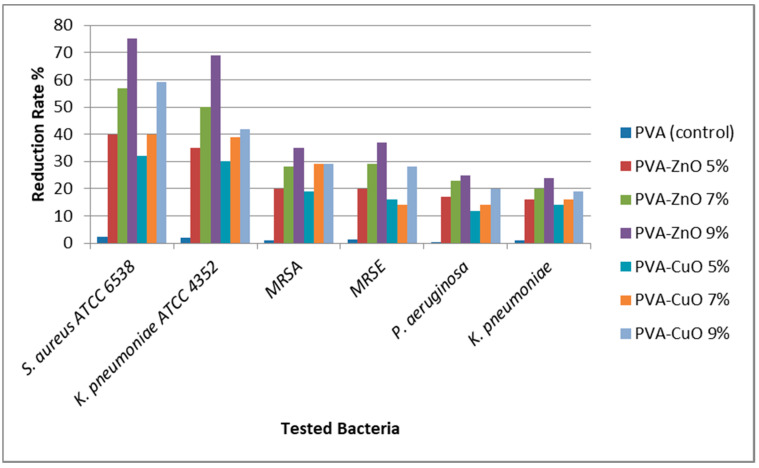
Reduction rate of the bacterial growth upon using the tested nanofibers, expressed in percentages.

**Figure 5:**
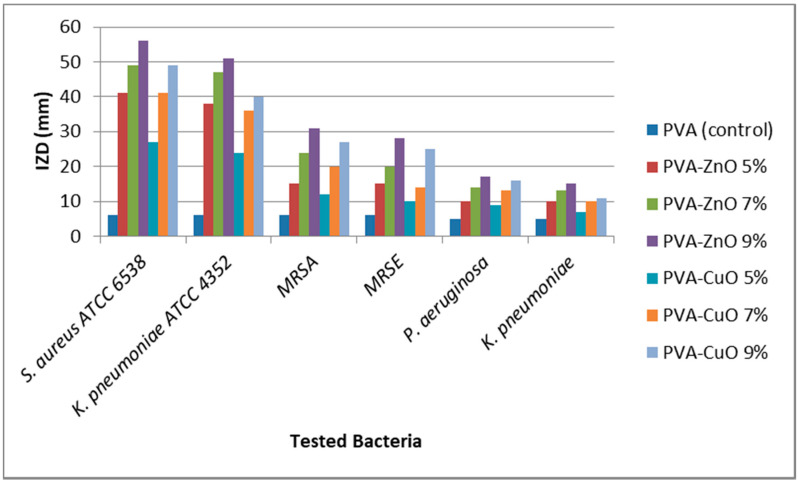
Inhibition zone diameter (IZD) of the tested nanofibers against ATTC and MDR bacteria.

Figure 4 and Figure 5 and Table 2 show the multi-drug resistant antibacterial activity of the developed nanofibers, evaluated by two techniques. Both techniques proved the superiority of the ZnO loaded nanofibers over the CuO loaded ones. It is known that metal oxide nanoparticles have a significant antibacterial activity due to several factors. The first is the small particle size, which helps the interaction between the oxides and the bacterial cells [29]. In the present study, ZnO loaded fibers had a slightly smaller particle size (210–250 nm) compared to the CuO loaded fibers (250–300 nm), which may explain the superior activity of ZnO. 

Nair et al. reported that zinc oxide nanoparticles showed a significant inhibitory effect against both Gram-negative and Gram-positive bacteria as compared to the bulk particles [30]. Similarly, antibacterial studies showed that the tested bacteria are highly affected by the size of the CuO nanoparticles [31].

Another factor affecting the metal oxide nanoparticle antibacterial activity is the metal oxide concentration, which proved to be a proportional relation (when the metal oxide nanoparticles concentration increases, the antibacterial activity increases). In the light of this, our results were proven to be in accordance with the aforementioned statement (by increasing the ZnO or CuO nanoparticle concentration, the antibacterial activity increased). 

The CuO bactericidal activity may be based on the ability of the released Cu^+^ ions to associate with thiol groups from amino acid residues in proteins, allowing them to easily penetrate bacterial cells and release copper ions more quickly after dissolution. Copper oxide’s antibacterial activity is highly dependent on the nanoparticle concentration in the test medium. Copper ions released from nanoparticles will electrostatically attract and rupture negatively charged cell envelope components, resulting in protein denaturation and cell death [32].

Since ZnO-NPs are less toxic to humans than CuO NPs and Ag NPs have a low cost of synthesis, they are commonly used in cosmetics products, medicines for wound healing, acne treatments, and fungal infection treatments [33,34]. The antibacterial mechanism of ZnO-NPs is wide-ranging and complex; the release of toxic dissolved Zn ions from ZnO-NPs results in a direct contact of ZnO-NPs with the bacterial cell envelope, which destroys the cell integrity and development of reactive oxygen species (ROS), consequently causing oxidative stress and cell wall damage, enhanced membrane permeability, and uptake of toxic dissolved Zn ions [35]. The released Zn from ZnO-NPs ions can have a significant impact on bacterial membrane permeability, as well as inactivating various biomolecules, especially enzymes, and cause cell death [31]. An alternative theory proposed that ZnO-NPs bind to the bacterial surface due to electrostatic forces, which destroy bacteria directly [32]. ZnO may also protect intestinal cells from *E. coli* infection by inhibiting bacterial adhesion and internalization by preventing the increase of tight junction permeability and modulating cytokine gene expression, according to a different protective mechanism proposed. With increasing nanoparticle concentrations, the amount of ROS produced from the surface of the ZnO should increase proportionally. The survival rate of bacteria decreases as the average concentration of reactive oxygen species (ROS) increases.

Norouzi et al. reported the synthesis of PVA/ZnO nanocomposite fibers with no cytotoxicity, and with highly efficient antibacterial and wound healing properties. The high concentration of TGF-β in the first days after injury resulted in increased angiogenesis and a faster wound healing process. According to a histological study, ZnO nanoparticles were responsible for accelerated epithelial regeneration and better cell attachment [36].

## 4. Conclusions

Nanofibrous membranes of polyvinyl alcohol (PVA) with ZnO and CuO nanoparticles were manufactured, characterized, and used to develop antiviral and antibacterial biodegradable membranes. Optimized spinning conditions with ZnO and CuO nanoparticles ratios were implemented, and a homogenous distribution and bead-free nanofibrous membranes were achieved in all samples. The results of antiviral and antibacterial activity against a variety of bacteria strains, including multi-drug resistant bacteria, as well as SARS-Cov-2, revealed ZnO loaded nanofibers to be superior as as antiviral and antibacterial layers than the copper ones. Recently, due to the COVID-19 pandemic, antimicrobial/antiviral fibrous products are expected to become heavily used in many applications, such as military uniforms, healthcare worker uniforms, workwear uniforms, household goods, and sports apparel. The developed nanofibrous membranes achieve the two main required and challenging properties, which are an anti-bacterial/antiviral activity and biodegradability. This makes the developed nanofibrous membranes promising functional layers for many types of personal protection equipment, such as masks, gowns, disposable bed sheets, etc.

## Figures and Tables

**Table 1:** Average Fiber Diameter (nm) of the developed nanofibers.

Nanofiber	Nanoparticles Concentration	Average Fiber Diameter (nm)
**PVA**	0%	260 ± 15
**PVA-ZnO**	5%	222 ± 20
	7%	261 ± 12
	9%	263 ± 15
**PVA-CuO**	5%	215 ± 10
	7%	285 ± 17
	9%	270 ± 22

**Table 2:** Multi-drug resistant antibacterial activity of the prepared nanofibers of PVA, PVA-ZnO, and PVA-CuO nanofibers with different concentrations.

Nanofiber	*S. aureus* ATCC 6538		*K. pneumoniae* ATCC 4352		MRSA **		MRSE ***		*P. aeruginosa*		*K. pneumoniae*	
	Reduction Rate %	IZD * (mm)	Reduction Rate %	*IZD* (mm)	Reduction Rate %	*IZD* (mm)	Reduction Rate %	*IZD* (mm)	Reduction Rate %	*IZD* (mm)	Reduction Rate %	*IZD* (mm)
PVA (control)	2.3	6	2	6	1.1	6	1.4	6	0.5	5	1	5
PVA-ZnO 5%	40	41	35	38	20	15	20	15	17	10	16	10
PVA-ZnO 7%	57	49	50	47	28	24	29	20	23	14	20	13
PVA-ZnO 9%	75	56	69	51	35	31	37	28	25	17	24	15
PVA-CuO 5%	32	27	30	24	19	12	16	10	12	9	14	7
PVA-CuO 7%	40	41	39	36	29	20	14	14	14	13	16	10
PVA-CuO 9%	59	49	42	40	29	27	28	25	20	16	19	11

*IZD* *: inhibition zone diameter, MRSA **: Methicillin resistant *S. aureus*, MRSE ***: Methicillin resistant *S. epidermidis*.

## Data Availability

Not applicable.

## Supplementary Materials

The following are available online at https://www.mdpi.com/article/10.3390/ma14143862/s1, Figure S1: TEM micrographs of ZnO NPs; Figure S2: TEM micrographs of CuO NPs; Figure S3: SEM micrographs of ZnO NPs; Figure S4: SEM micrographs of CuO NPs; Figure S5: XRD pattern of ZnO NPs; Figure S6: XRD pattern of CuO NPs; Figure S7: FTIR spectra of the ZnO NPs; Figure S8: FTIR spectra of the CuO NPs.
