# Supplementary material for: Biodegradable Nanofibrous Membranes for Medical and Personal Protection Applications: Manufacturing, Anti-COVID-19 and Anti-Multidrug Resistant Bacteria Evaluation
Source: Materials (Basel). 2021 Jul 10;14(14):3862. doi: 10.3390/ma14143862 (PMC8306818; doi:10.3390/ma14143862)
Supplement: Supplementary file 1 [file materials-14-03862-s001.zip › materials-1263528-supplementary.pdf]

materials

MDPI

Article

Biodegradable Nanofibrous Membranes for Medical and Personal Protection Applications: Manufacturing, Anti-COVID-19 and Anti-Multidrug Resistant Bacteria Evaluation

Latifah Abdullah Alshabanah [1], Mohamed Hagar [2,3,*], Laila A. Al-Mutabagani [1], Ghada M. Abozaid [4], Salwa M. AbdAllah [5], Hoda Ahmed [2,6], Ahmed H. Hassanin [7,8,9] and Nader Shehata [7,10,11,12]

[1] Chemistry Department, College of Science, Princess Nourah Bint Abdulrahman University, Riyadh 11671, Saudi Arabia; laalsabanah@pnu.edu.sa (L.A.A.); laalmutbagani@pnu.edu.sa (L.A.A.-M.)
[2] Chemistry Department, College of Sciences, Taibah University, Yanbu 30799, Saudi Arabia; ahoda@sci.cu.edu.eg
[3] Chemistry Department, Faculty of Science, Alexandria University, Alexandria 21321, Egypt
[4] Pharmaceutical Practice Department, College of Pharmacy, Princess Nourah Bint Abdulrahman University, Riyadh 11671, Saudi Arabia; Gaabozeed@pnu.edu.sa
[5] Mammalian and Aquatic Toxicology Department, Central Agricultural Pesticides Lab (CAPL), Agricultural Research Center (ARC), Giza 12611, Egypt; salwaabdallah17@gmail.com
[6] Department of Chemistry, Faculty of Science, Cairo University, Cairo 12613, Egypt
[7] Center of Smart Materials Nanotechnology and Photonics (CSMNP), Smart CI Research Centre, Alexandria University, Alexandria 21544, Egypt; ahassanin2003@yahoo.com (A.H.H.); nader83@vt.edu (N.S.)
[8] Materials Science & Engineering Department, School of Innovative Design Engineering, Egypt-Japan University of Science and Technology (E-JUST), New Borg El-Arab City, Alexandria 21934, Egypt
[9] Department of Textile Engineering, Faculty of Engineering, Alexandria University, Alexandria 21544, Egypt
[10] Department of Engineering Mathematics and Physics, Faculty of Engineering, Alexandria University, Alexandria 21544, Egypt
[11] USTAR Bio Innovations Centre, Faculty of Science, Utah State University, Logan, UT 84341, USA
[12] Department of Physics, School of Engineering, Kuwait College of Science and Technology (KCST), Doha Superior Rd., Jahraa 13133, Kuwait
* Correspondence: mhagar@taibahu.edu.sa

Transmission Electron Microscope Images (TEM)

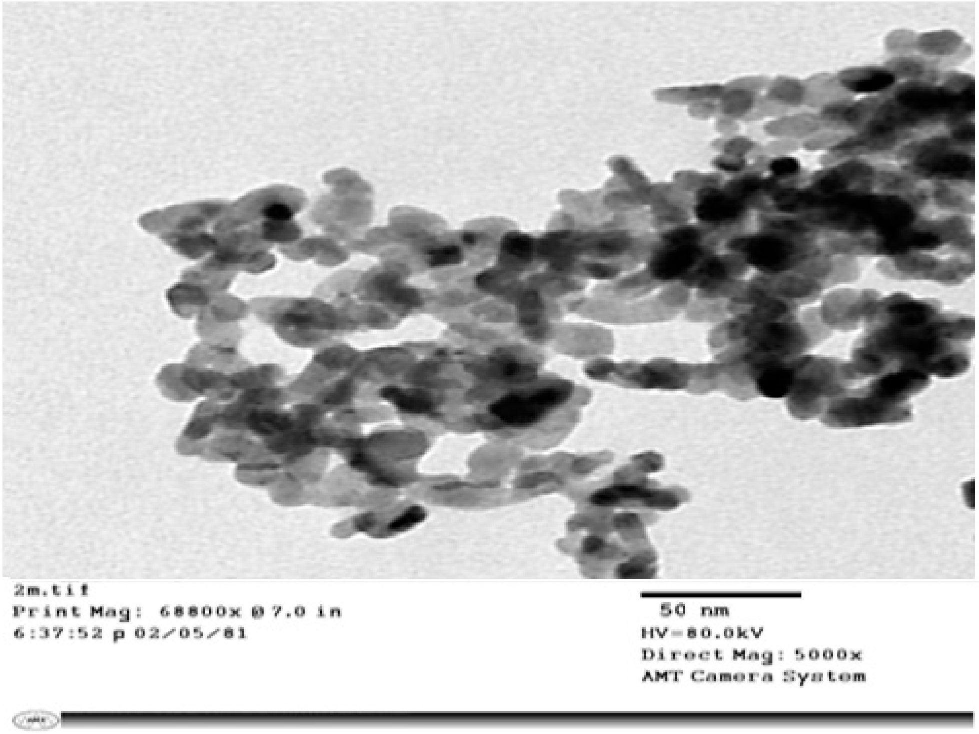

Figure S1. TEM micrographs of ZnO NPs.

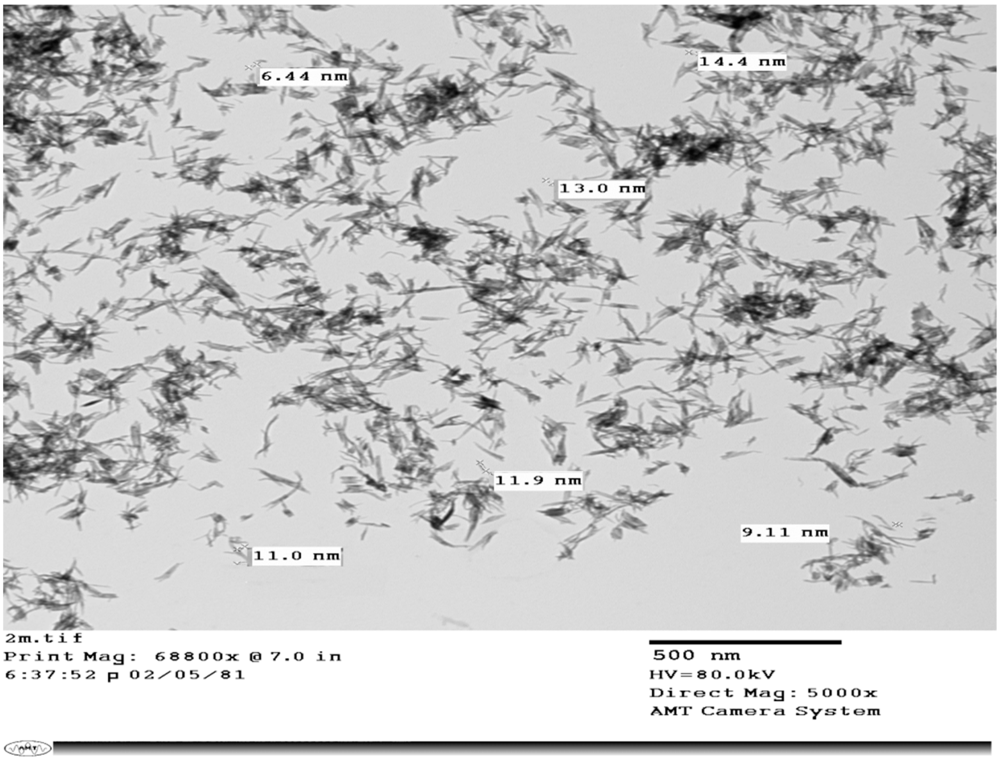

Figure S2. TEM micrographs of CuO NPs.

1.2- SEM analysis:

The SEM images of ZnO samples show that the agglomerations of particles are much less in this method of preparation.

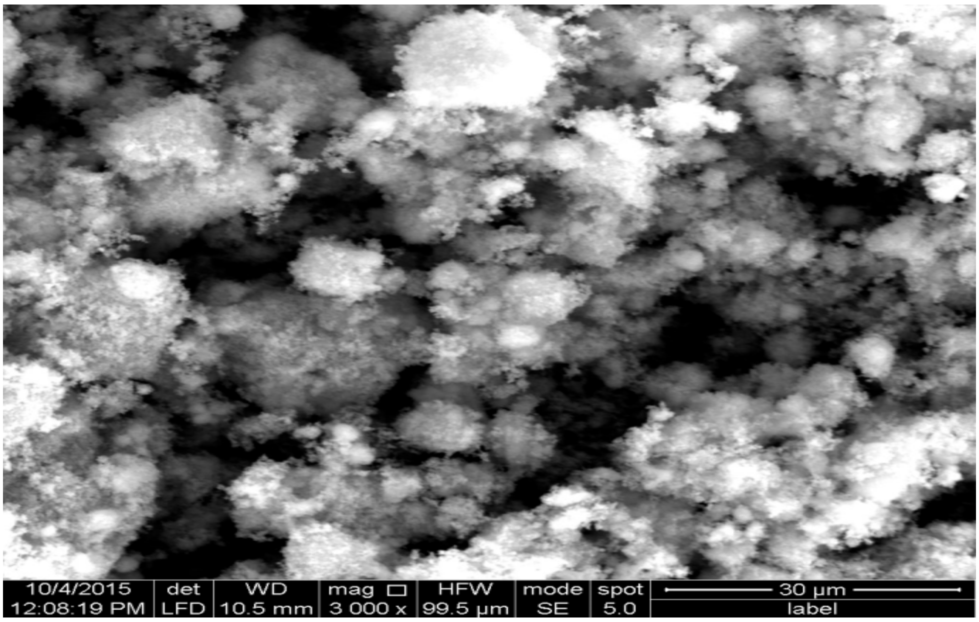

Figure S3. SEM micrographs of ZnO NPs.

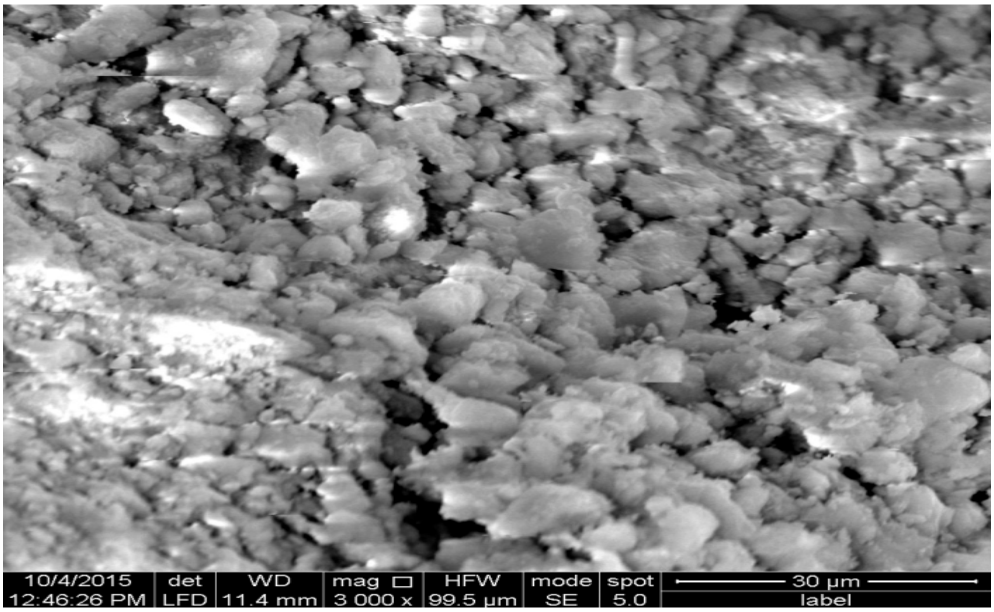

Figure S4. SEM micrographs of CuO NPs.

1.3-X-ray Diffractometer Analysis:

XRD pattern of ZnO NPs indicated only characteristic peaks for crystalline ZnO hexagonal phase at (31.74°, 34.42°, 36.24°, 47.5°, 56.62°, 62.83°, 66.4°, 67.93°, 69.05°) respectively were indexed to (100), (002), (101), (102), (110), (103), (200), (112) and (201) planes. All the diffraction peaks are well indexed to the hexagonal ZnO wurtzite structure (JCPDS no. 36–1451). Diffraction peaks corresponding to the impurity were not found in the XRD patterns, confirming the high purity of the synthesized products.

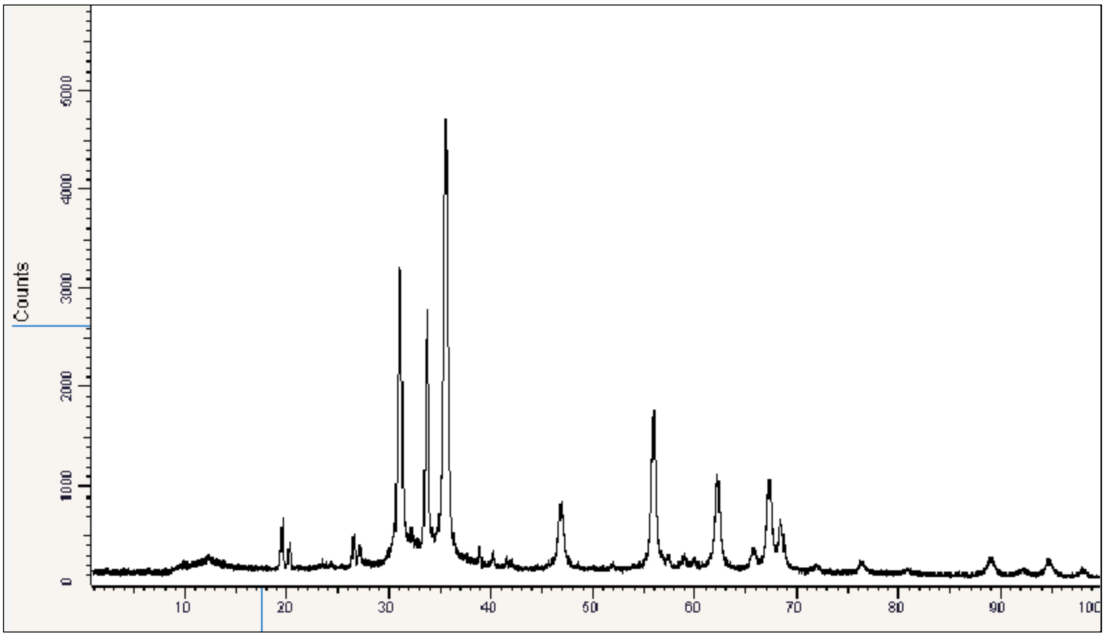

Figure S5. XRD pattern of ZnO NPs.

Crystalline nature of the prepared CuO nanoparticles was identified from their corresponding powder XRD patterns (Figure 20). All the diffractions were well matched with monoclinic phase of CuO (standard JCPDS File No: 048-1548). Diffraction peaks with 2Ɵ 33.6°, 35.45°, 38.73°, 48.92°, 61.99° and 66.49° respectively were indexed to (110), (002), (111), (020), (022) and (113) planes.

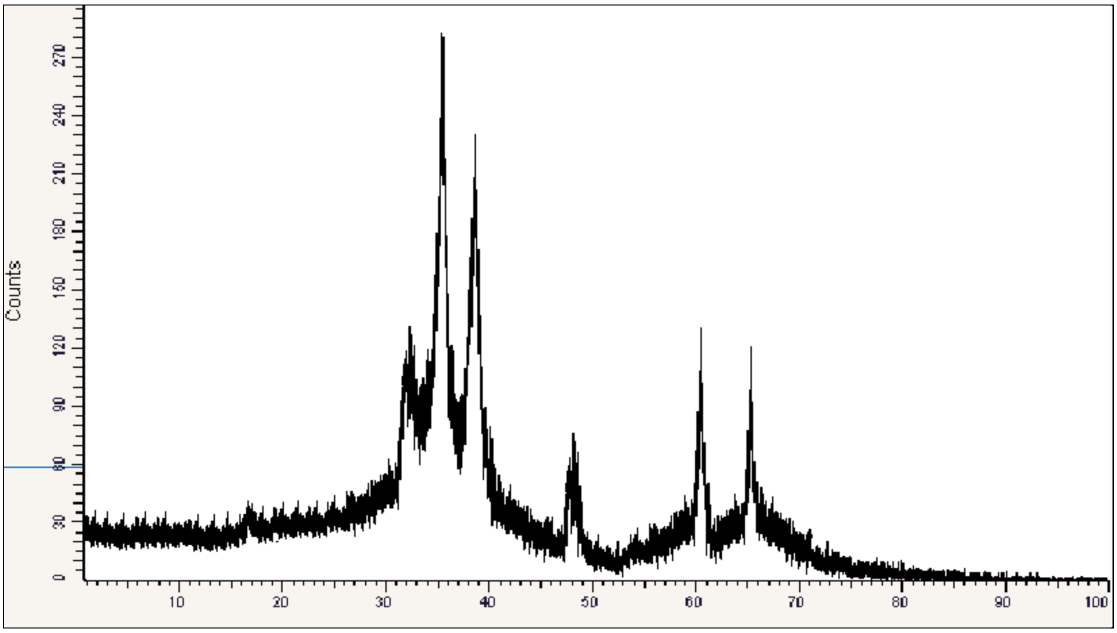

Figure S6.XRD pattern of CuO NPs.

1.5- FT-IR spectroscopy Analysis:

Figure S7 showed the FTIR spectra of ZnO nanoparticles. Metal oxides generally give absorption bands in finger print region i.e. below 1000 cm^{-1} arising from inter-atomic vibrations. The peak at 1388.70 cm^{-1} and 1507.80 cm^{-1} corresponds to C=O and C-O bending

vibrations respectively .The peak observed at 3366.35 and 1036.07 cm^{-1} are may be due to O-H stretching and deformation, respectively assigned to the water adsorption on the metal surface.

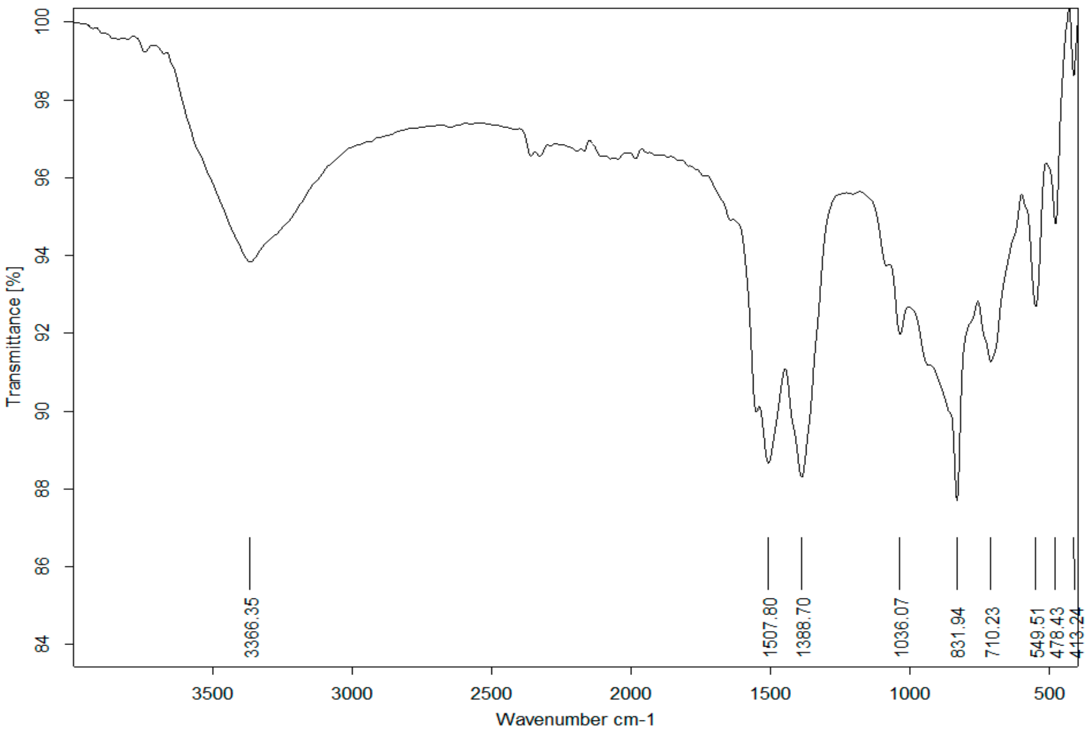

Figure S7. FTIR spectra of the ZnO NPs.

Figure S8 showed the FTIR spectra of CuO-NPs. The broad absorption peak at 3390 cm^{-1} was caused by the adsorbed water molecules. Since the nano crystalline materials possess a high surface to volume ratio, they can absorb moisture. The peaks at 1633 may be for the Cu-O symmetrical stretching. The high-frequency mode at 605.05 cm^{-1}, 490.60 cm^{-1} can be assigned to the Cu-O stretching vibration. Moreover, no other IR active mode was observed in the range of 605 to 660 cm^{-1}, which totally rules out the existence of another phase, i.e., Cu_2O. Thus, the pure phase CuO with monoclinic structure is also confirmed from the FTIR analysis.

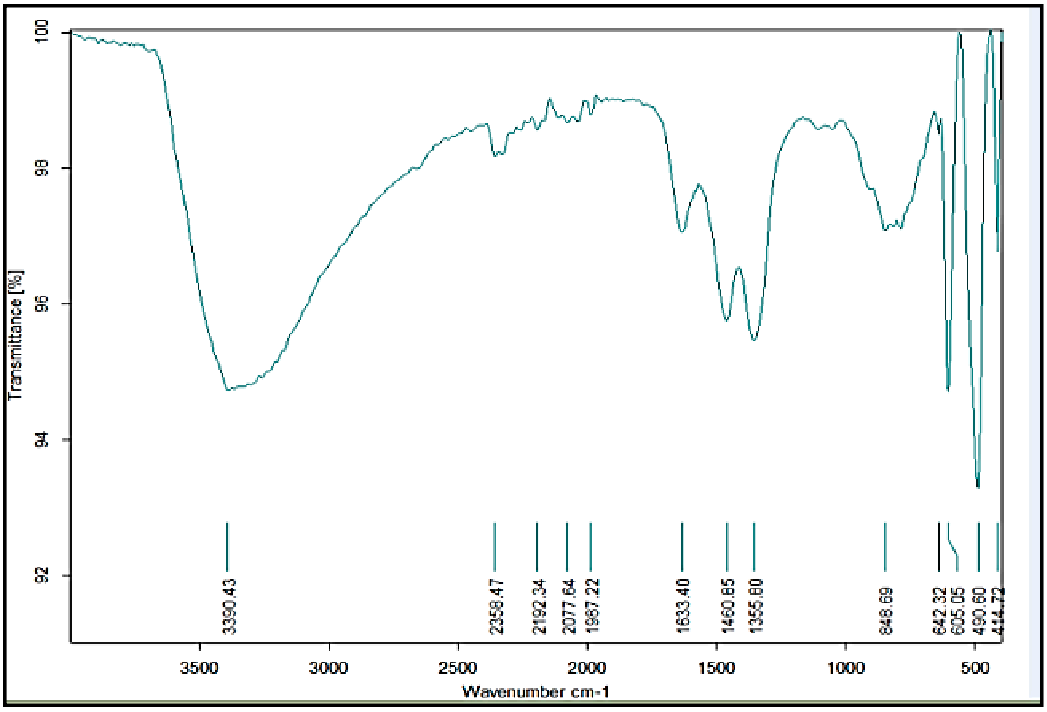

FigureS8. FTIR spectra of the CuO NPs.
